# An implicit and reliable neural measure quantifying impaired visual coding of facial expression: evidence from the 22q11.2 deletion syndrome

**DOI:** 10.1038/s41398-019-0411-z

**Published:** 2019-02-04

**Authors:** Arnaud Leleu, Emilie Favre, Alexandre Yailian, Hugo Fumat, Juliette Klamm, Isabelle Amado, Jean-Yves Baudouin, Nicolas Franck, Caroline Demily

**Affiliations:** 1 Developmental Ethology and Cognitive Psychology group, Centre des Sciences du Goût et de l’Alimentation, CNRS, Université Bourgogne Franche-Comté, Inra, AgroSup Dijon, F-21000 Dijon, France; 2 Reference Center for Rare Diseases with Psychiatric Phenotype - GénoPsy, Centre Hospitalier le Vinatier, Marc Jeannerod Institute (CNRS & Claude Bernard Lyon 1 University), Bron, France; 3 Child and Adolescent Psychiatry, University Hospital of Montpellier, University Montpellier 1, Montpellier, France; 4 Centre Ressource de Réhabilitation Psychosociale et de Remédiation Cognitive, Centre Hospitalier Le Vinatier & Université Lyon 1 (CNRS UMR 5229), Université de Lyon, Lyon, France; 5 Centre Ressource Ile de France de Remédiation Cognitive et Réhabilitation Psychosociale, Groupe Hospitalier Universitaire, Institut de Psychiatrie et Neurosciences de Paris, Université Paris Descartes, Paris, France; 6 Laboratoire Développement, Individu, Processus, Handicap, Éducation (DIPHE), Département Psychologie du Développement, de l′Éducation et des Vulnérabilités (PsyDEV), Institut de Psychologie, Université de Lyon (Lumière Lyon 2), 69676 Bron Cedex, France

## Abstract

Although various psychiatric disorders present with social-cognitive impairment, a measure assessing social-cognitive processes implicitly and reliably, with high selectivity and with enough signal-to-noise ratio (SNR) for individual evaluation of any population at any age, is lacking. Here we isolate a neural marker quantifying impaired visual coding of facial expression in individuals with 22q11.2 deletion syndrome (22q11DS) using frequency-tagging with electroencephalography (EEG). Twenty-two 22q11DS participants and 22 healthy controls were presented with changes of facial expression displayed at low, moderate, and high intensities every five cycles in a stream of one neutral face repeating 6 times per second (i.e., at a 6 Hz base rate). The brain response to expression changes tagged at the 1.2 Hz (i.e., 6 Hz/5) predefined frequency was isolated over occipito-temporal regions in both groups of participants for moderate- and high-intensity facial expressions. Neural sensitivity to facial expression was reduced by about 36% in 22q11DS, revealing impaired visual coding of emotional facial signals. The significance of the expression-change response was estimated for each single participant thanks to the high SNR of the approach. Further analyses revealed the high reliability of the response and its immunity from other neurocognitive skills. Interestingly, response magnitude was associated with the severity of positive symptoms, pointing to a potential endophenotype for psychosis risk. Overall, the present study reveals an objective, selective, reliable, and behavior-free signature of impaired visual coding of facial expression implicitly quantified from brain activity with high SNR. This novel tool opens avenues for clinical practice, providing a potential early biomarker for later psychosis onset and offering an alternative for individual assessment of social-cognitive functioning in even difficult-to-test participants.

## Introduction

Social-cognitive impairment is a hallmark characteristic of various psychiatric disorders, such as schizophrenia^1,2^, autism spectrum disorders^3^, and genetic syndromes associated with broad psychiatric phenotypes^4^. Precisely delineating the mechanisms underlying impaired social cognition in these populations and developing validated tools to assess their social-cognitive processes is a major challenge to better understand the neurocognitive determinants of inappropriate social skills and more generally to promote early clinical interventions. Our aim here was therefore to establish the proof-of-concept for a selective measure of the visual coding of facial expression—a key component of social cognition—that could be usable in a clinical context to assess any population at any age.

For this purpose, we focused on the 22q11.2 deletion syndrome (22q11DS), a genetic condition affecting up to 1 per 2000 individuals^5^ and one of the highest known risks for schizophrenia spectrum disorders (SSD) with approximately 40% of the affected people having developed psychosis in adulthood^6^. Reduced social functioning and social-cognitive impairment are well documented in 22q11DS^7^, both associated with psychotic symptoms^8–14^, and with neural underpinnings in the structural integrity of the “social brain network”^15–17^. In particular, a large body of research investigated the recognition of emotions from facial expression and reported that 22q11DS individuals exhibit difficulties to discriminate or identify facial emotional signals^9,12–14,18–26^. Impaired detection of facial emotions expressed at variable intensities^14,22,25,27^ and abnormal visual exploration of expressive faces^18,20,23,27^ were also described.

However, discrepancies remain across studies about the most relevant measure of impaired facial emotion recognition (e.g., accuracy vs. reaction times^13,14,21,24,27^), the differences between 22q11DS and control participants in visual exploration patterns for expressive faces^18,20,23,27^, or the selectivity of the observed difficulties (refs. ^12,20,22,23,27^ and refs. ^25,26^, respectively, for the contribution of general cognitive functioning and basic visual processing). These inconsistent findings may be explained by the use of explicit behavioral responses from different methodologies tapping various mechanisms with variable sensitivity. Especially, response-related processes (e.g., working memory, response inhibition) are conflated with the visual coding of facial expression in explicit behavioral measures^28^ and easily contaminated by comprehension, motivation, and attention. Hence, the contribution of such unspecific processes cannot be definitely ruled out from explicit behavioral outputs in 22q11DS individuals suffering from verbal difficulties^29–32^, impaired executive processes^24,30,33–35^, low attentional resources^29,30^, and reduced motivation^36^. Moreover, explicit measures also require variable age-appropriate testing tools that hinder the opportunity to develop life-span standardized assessments in 22q11DS and more generally in any other neurodevelopmental disorder.

Neural measures are ideally suited to provide alternative biomarkers for unambiguous quantification of the visual coding of facial expression in any population at any age. Using orthogonal behavioral tasks (i.e., implicit processing of facial expression), functional neuroimaging studies^37–39^ revealed abnormal sensitivity to facial expression in 22q11DS within visual brain regions from the core cortical network mediating face perception^40^ where structural alterations have been reported^41–44^. Interestingly, normal activation when viewing houses as well as lower sensitivity to faces in psychotic vs. non-psychotic participants^38^ suggest a face-specific visual impairment eligible as an endophenotype for psychosis risk in 22q11DS.

However, as for behavioral measures, findings lack consistency across studies (e.g., higher vs. equal vs. lower expression-selective activation in the fusiform gyrus for 22q11DS compared with healthy participants^37–39^). Unfortunately, functional neuroimaging exhibits technical limitations that hamper its potential translation into clinical practice. It requires long recording sessions to reach adequate signal-to-noise ratios (SNRs) that prevent from rapid testing and individual assessment. In addition, analysis routines are not standardized across studies, reducing objectivity and generalizability. When a behavioral task is performed during stimulation, even an orthogonal task, response-related processes can still contaminate the recorded neural response. Finally, standard post hoc subtraction contrasting expressive from neutral faces indirectly reflects their visual discrimination^45,46^. Hence, neuroimaging remains limited for developing an implicit and objective measure that rapidly isolates the visual coding of facial expression with high SNR and selectivity in any individual participant.

Here we circumvent these issues and provide a neural marker of impaired visual coding of facial expression in 22q11DS individuals using a frequency-tagging approach^47^ with scalp electroencephalography (EEG). Previous frequency-tagging studies in healthy individuals quantified a neural response to facial expression change in a few minutes in every participant with consistent response patterns across studies^48,49^. Accordingly, following ref. ^49^, 22q11DS and healthy participants performed a non-periodic orthogonal task while presented with a neutral face at a rapid 6 Hz rate and brief changes of facial expression inserted every fifth cycle (i.e., at a 6/5 = 1.2 Hz rate) at low, moderate, and high intensities. Using a frequency-domain analysis of EEG amplitude spectra, the visual discrimination of facial expression was objectively measured at the predefined 1.2 Hz frequency with high SNR and directly dissociated from general unspecific visual processes captured at the 6 Hz rate of stimulus presentation within the same stimulation sequence (i.e., without post hoc subtraction).

## Materials and methods

### Participants

Twenty-two individuals with 22q11.DS (10 females, mean age: 25.3 ± 8.7 (SD) years, range 16–44 years) and 22 healthy participants (7 females, mean age: 26.9 ± 6.7 years, range 19–42 years) participated in the study (sample size in adequacy with previous studies showing impaired recognition of facial expression in 22q11DS). The two groups did not differ according to age (*T*_42_ = 0.72, *p* = .48) nor sex (*X*^2^_1_ = 0.86, *p* = .35). Participants with 22q11DS were diagnosed using comparative genomic hybridization array analysis confirmed by fluorescence in situ hybridization analysis. Eight of them presented concomitant diagnosis of schizophrenia (Diagnostic and Statistical Manual of Mental Disorders, Fifth Edition (DSM-5) criteria) and were treated with antipsychotic medication. All were clinically stable at testing time with unchanged medication during the month preceding the experiment. Detailed information about 22q11DS participants at their inclusion is available in Supplementary Table S1. Healthy participants did not have any relative with 22q11DS. Exclusion criteria were pregnancy, substance abuse (DSM-5 criteria except for tobacco and caffeine), neurodegenerative, neurovascular, infectious disorders or head injury, uncorrected loss of visual acuity, brain stimulation during the 2 previous months, or inclusion in a social-cognitive remediation program. Absence of premorbid intellectual disability was confirmed using the French adaptation of the National Adult Reading Test^50^. The study was conducted according to the Declaration of Helsinki and approved by the French ethics committee (Comité de protection des personnes Sud-Est II - ANSM Reference:151109B-31; ID RCB: 2015-A01247-42). All participants (parents for minor participants) provided written informed consent before inclusion. Participants with 22q11DS completed clinical and neuropsychological evaluations. Clinical ratings were obtained using the positive and negative symptoms scale^51^, a reliable measurement of psychotic symptoms largely used in psychiatry research, e.g., ref. ^52^. Neuropsychological assessment evaluated various cognitive processes detailed in Supplementary Methods. Results for both clinical and neuropsychological assessments are summarized in Supplementary Table S3 and discussed in Supplementary Results.

### Stimuli

**Fig. 1:**
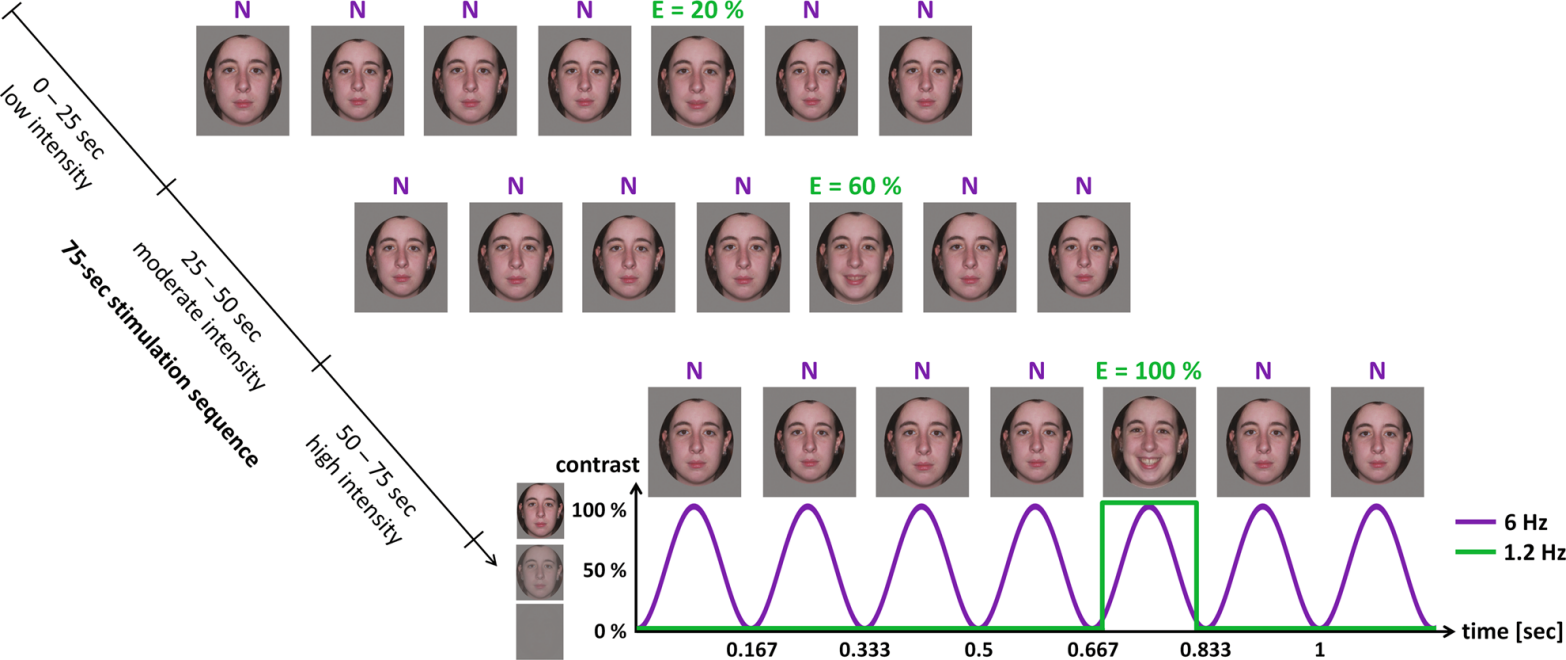
A frequency-tagging approach in electroencephalography (EEG) isolating a selective brain response to brief changes of facial expression. During stimulation, an individual neutral face (N) is presented through sinusoidal contrast modulation at a rapid rate of 6 Hz (one stimulus lasts ≈167 ms). The same face expressing an emotion (E) among the five tested (e.g., happiness) is inserted every fifth stimulus, introducing a brief change of expression at a rate of 6/5 = 1.2 Hz (≈833 ms between two expression changes). Stimulus size varies randomly between 95% and 105% at every stimulus onset. Each stimulation sequence lasts 75 s with expression intensity increasing every 25 s throughout the sequence. During the first 25 s, emotions are expressed at low intensity (20% of the full-blown expression), then at moderate (i.e., 60%) intensity during the next 25 s, and finally at high (i.e., 100%) intensity during the last 25 s. Two distinct neural responses are thus dissociated in the EEG frequency spectrum: a general visual response at 6 Hz and harmonics (i.e., integer multiples) reflecting the processing of all cues (e.g., contrast) that rapidly flicker at 6 Hz; an expression-change response at 1.2 Hz and harmonics selectively indexing the visual coding of facial expression against neutrality

Face stimuli were adapted from previous studies^25,49^ and comprised 24 color pictures from 4 individuals (2 females) with a neutral expression or expressing 5 basic emotions (anger, disgust, fear, happiness, sadness) in full-front view. Faces were cropped into medallion-shaped windows displayed on a mid-level gray (i.e., 128/255 in grayscale) background (Fig. 1 and Supplementary Movie). For each individual face and emotional expression, 3 intensity levels (i.e., low = 20% of the full-blown expression, moderate = 60%, high = 100%) were designed using a morphing procedure (Morpheus Photo Morpher 1.85, Morpheus Software, USA) combining neutrality and the expression. The final set of stimuli was thus composed of 64 pictures, 16 for each individual face (3 intensities × 5 emotions + neutrality).

### Procedure

The procedure was adapted from previous studies using frequency-tagging with EEG to investigate facial expression perception^48,49^. Stimuli were displayed on a computer screen (60 Hz refresh rate) with a mid-level gray background (i.e., 128/255 in grayscale) through sinusoidal contrast modulation (from 0% to 100%) at a rapid rate of 6 Hz (Fig. 1 and Supplementary Movie) using a custom software written in Java. At this rate, each stimulus lasts ≈167 ms with full contrast reached around 83 ms. Neutral faces were presented at the 6 Hz base rate and an emotional expression was introduced every fifth stimulus. Thus a brief change of expression from neutrality to an emotion occurred at a rate of 6/5 = 1.2 Hz (i.e., ≈833 ms between two expressive faces). To reduce expression-change detection based on low-level visual cues, stimulus size was randomly varied between 95% and 105% at every stimulus onset (initial size was set to 6 × 4.8° of visual angle when displayed on the screen).

Several stimulation sequences were presented with one individual neutral face changing to one emotional expression throughout each sequence. Sequences started with a variable interval of 0.5–1 s of blank screen, followed by a 2.5-s fade-in of increasing contrast modulation depth with no expression change. Then stimulation lasted 75 s with intensity of the 1.2 Hz expression change increasing every 25 s (i.e., during the first 25 s, the emotion was expressed at low (20%) intensity, followed by the moderate (60%) intensity during the next 25 s, finally followed by the high (100%) intensity during the last 25 s). A 1-s fade-out of decreasing contrast modulation depth then followed and sequences ended with a variable interval of 0.5–1 s of blank screen. Each emotion was repeated 4 times (4 individual faces), resulting in 20 sequences throughout the experiment. They were divided into 4 blocks of 5 sequences, each block presenting one sequence per emotion. The presentation orders of blocks and sequences within blocks were randomized across participants.

Participants were seated in front of a computer screen at a distance of 47 cm. To maintain their attention on the screen, they performed an orthogonal behavioral task. During each sequence, a fixation circle (located just below the eyes when the face stimuli were displayed) briefly (i.e., 200 ms) changed into a square 9 random times with a minimum interval of 5 s between two changes. Participants were asked to detect these shape changes by pressing the space bar of a keyboard as fast as possible. Response times and accuracy are detailed in Supplementary Results.

### EEG acquisition and analysis

Throughout the experiment, EEG was continuously recorded from 128 Ag/AgCl electrodes mounted in an electrode cap (Waveguard, ANT, The Netherlands) according to the 10–10 classification system (acquisition reference: AFFz, electrode impedance <15 kΩ, sampling rate: 1024 Hz). EEG data were then preprocessed and averaged across individual faces for each facial expression. Primary analyses in the frequency domain (Supplementary Methods) were performed to isolate and quantify both brain responses to the 6 Hz stimulation rate and to the 1.2 Hz expression-change rate and their harmonics (i.e., integer multiples). Note that frequency-domain analysis allows high SNR measurement since the signal is captured in tiny target frequencies (e.g., 1.2 Hz, 2.4 Hz, etc.) while the noise spreads to broad frequency ranges. Here frequency-domain resolution was 0.04 Hz, leading to 29 frequency bins between two target frequencies (e.g., between 1.2 Hz and 2.4 Hz). These surrounding frequency bins were used for noise level estimation. Statistical analyses were finally conducted using *Z*-scores (to estimate the significance of each brain response compared with surrounding noise level, see Supplementary Methods) and repeated-measures analyses of variance (ANOVAs) performed on EEG amplitudes corrected for surrounding noise level. ANOVAs included *Group* (22q11DS, controls) as a between-subject factor and *Emotion* (anger, disgust, fear, happiness, sadness) and *Intensity* (low, moderate, high) as within-subject factors. ANOVAs also included regions of interest (*ROIs*) as a within-subject factor. Four ROIs (left, medial, and right occipital [lO, mO, and rO, respectively], and medial parietal [mP]) were analyzed for the response elicited by the 6 Hz stimulation rate, and two ROIs (left and right occipito-temporal [lOT and rOT, respectively]) for the response elicited by the 1.2 Hz expression-change rate (see Supplementary Methods for definition of ROIs). Mauchly’s test for sphericity violation was computed and Greenhouse-Geisser correction for degrees of freedom was applied whenever sphericity was violated. For significant effects involving the *Group* factor, comparisons between groups of participants were conducted using *T* tests. Pearson’s correlation coefficients were calculated to explore the association between each brain response and both clinical and neuropsychological assessments, as well as the reliability of the expression-change response.

## Results

For both groups of participants, the 6 Hz base rate of rapid stimulation and the 1.2 Hz rate of brief expression change elicited periodic EEG responses identified in the frequency domain at the same frequencies and their harmonics. Detailed description of the EEG amplitude spectra obtained for both brain responses and their harmonics is available in Supplementary Results. Here we present summed corrected amplitudes for significant harmonics (Supplementary Methods), for they summarize each response in a single value expressed in microvolts (µV). For sake of brevity and according to our main purpose, we report only the expression-change response as a direct signature of the visual system ability to process expressive faces (results for the general visual response elicited by the 6 Hz stimulation rate are detailed in Supplementary Results). Similarly, despite some brief general descriptions of the response, we focus on statistical effects involving the *Group* factor to characterize the visual coding of facial expression in 22q11DS participants as compared with healthy controls.

### Impaired visual coding of moderate- and high-intensity facial expressions

As previously observed^49^, introducing brief changes of facial expression in a rapid sequence of neutral faces does not elicit identifiable brain activities at low expression intensity (*M* = 0.03 ± 0.02 (SEM) µV) while an occipito-temporal response progressively emerges as expression intensity increases (moderate intensity: *M* = 0.26 ± 0.03 µV, high intensity: *M* = 0.62 ± 0.06 µV, *Intensity* effect: *F*_1.4, 56.9_ = 84.66, *ε* = 0.68, *p* < .001, *η*_p_^2^ = 0.67). This response is larger in the right hemisphere (*ROI* effect: *F*_1, 42_ = 8.49, *p* = .006, *η*_p_^2^ = 0.17) with a greater amplitude increase as a function of expression intensity (high minus low intensity: *M* = + 0.64 ± 0.07 µV) than in the left hemisphere (*M* = + 0.54 ± 0.06 µV, *Intensity* × *ROI*: *F*_2, 84_ = 3.52, *p* = .034, *η*_p_^2^ = 0.08).

**Fig. 2:**
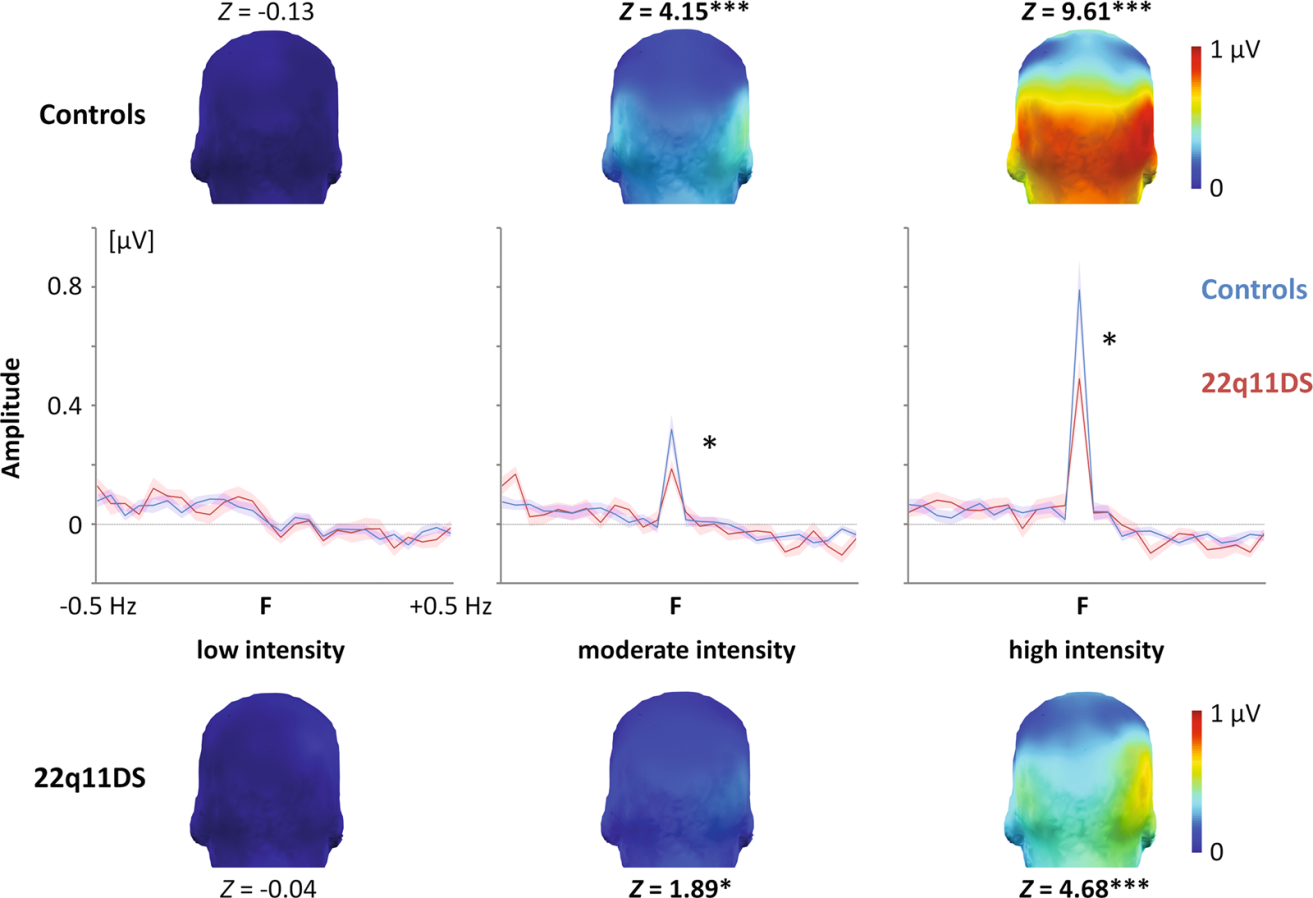
The expression-change brain response in both healthy and 22q11DS participants. Three-dimensional topographical maps (posterior view) of summed corrected amplitudes of the expression-change response as a function of expression intensity averaged across emotions for both healthy controls (top) and 22q11DS individuals (bottom). Center: the same data displayed averaged across regions of interest (ROIs) showing the expression-change response (F) surrounded by ±0.5 Hz of summed corrected amplitude spectra. Shadowed areas represent standard errors of the mean. *Z*-scores displayed next to each map indicate the significance of the response for each group and each expression intensity (*p < .05, ***p < .001). While the expression-change response is non-significant and practically absent for both groups of participants when emotions are expressed at low intensity, it progressively emerges as expression intensity increases with significant responses for both groups at moderate and high intensities. However, it is significantly lower in 22q11DS participants with a magnitude corresponding to 64% of the response observed for healthy controls

Most importantly for our purpose, a main effect of *Group* (*F*_1, 42_ = 5.01, *p* = .031, *η*_p_^2^ = 0.11) qualified by a *Group* × *Intensity* interaction (*F*_1.4, 56.9_ = 4.79, *ε* = 0.68, *p* = .023, *η*_p_^2^ = 0.10) revealed that while the expression-change response is almost absent at low expression intensity for both groups of participants (22q11DS: *M* *=* 0.03 ± 0.04 µV, controls: *M* = 0.02 ± 0.02 µV) with no difference between groups (*p* = .75), the increase of the response as a function of expression intensity is lower in 22q11DS individuals (*M* = + 0.45 ± 0.06 µV) than healthy controls (*M* = + 0.73 ± 0.09 µV) with significant differences between groups at both moderate (22q11DS: *M* = 0.20 ± 0.03 µV, controls: *M* = 0.32 ± 0.05 µV, *p* = .044) and high (22q11DS: *M* = 0.48 ± 0.06 µV, controls: *M* = 0.75 ± 0.09 µV, *p* = .021) intensities (Fig. 2). For both groups of participants, *Z*-scores confirmed the lack of expression-change response when emotions are expressed at low intensity (22q11DS: *Z* *=* −0.04, controls: *Z* = −0.13, both *p*s > .51, one-tailed, signal > noise) while emerging at moderate (22q11DS: *Z* = 1.89, *p* = .029; controls: *Z* = 4.15, *p* < .001) and high (22q11DS: *Z* = 4.68, controls: *Z* = 9.61, both *p*s < .001) intensities (Fig. 2). In sum, the visual coding of periodic expression changes inserted in a rapid sequence of neutral faces is impaired in individuals with 22q11DS compared with healthy participants, leading to a reduction of about 36% of the “typical” expression-change response when emotions are expressed at moderate and high intensities. Note that a significant *Group* × *Emotion* interaction (*F*_4, 168_ = 2.65, *p* = .035, *η*_p_^2^ = 0.06) revealed that amplitude differences between groups are not equivalent for every facial emotion, but this observation did not survive when controlling for the effects of antidepressant and antipsychotic medications (Supplementary Results).

### Individual brain responses to brief changes of facial expression

**Fig. 3:**
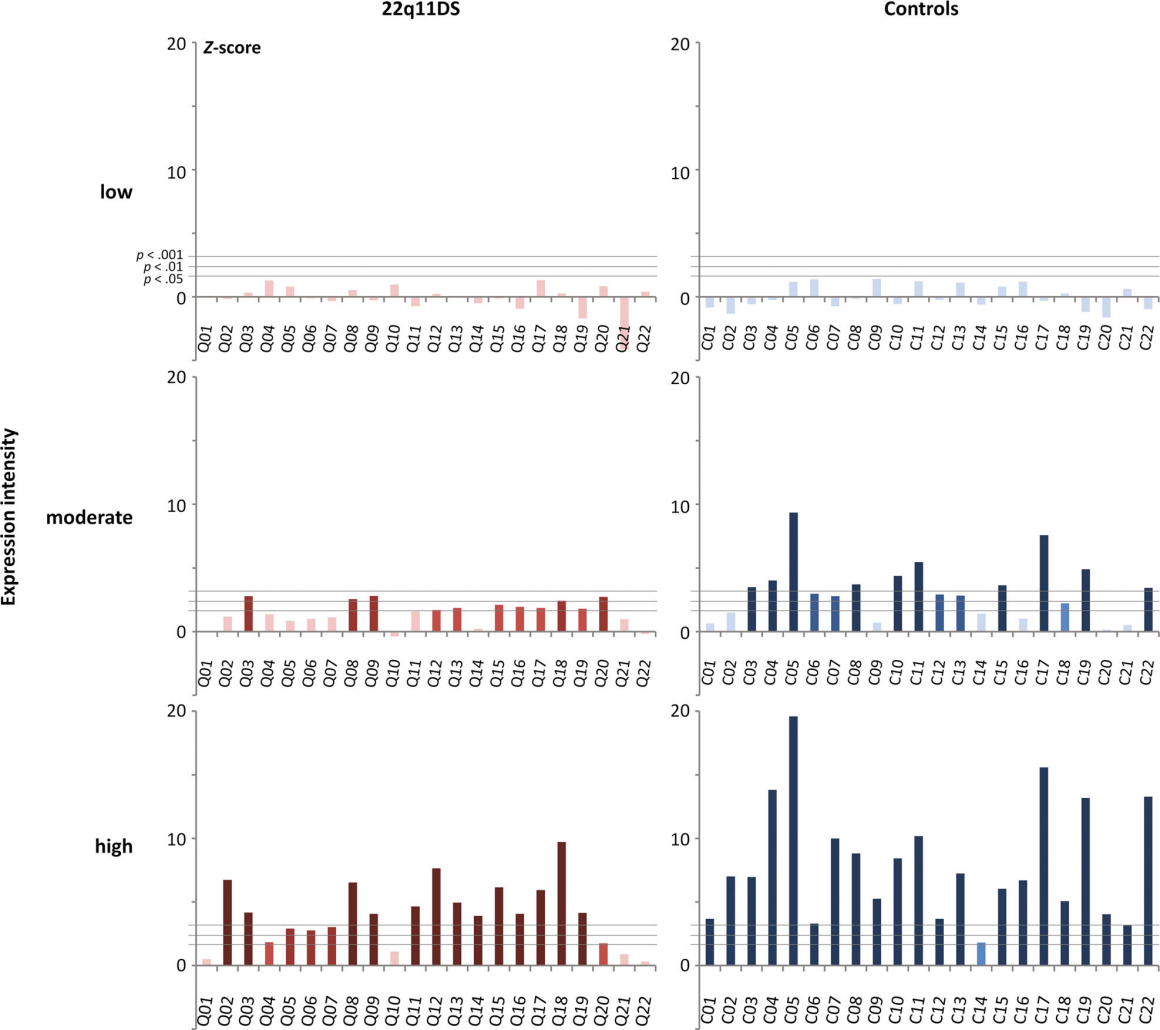
Individual brain responses to brief changes of facial expression. *Z*-scores estimating the significance of the expression-change response for every single participant from both the 22q11DS (left) and the control (right) groups and for low (top), moderate (center), and high (bottom) expression intensities averaged across emotions and regions of interest (ROIs). In each graph, horizontal gray bars indicate three significance thresholds (*Z* > 1.64, *p* < .05, one-tailed, signal > noise; *Z* > 2.32, *p* < .01; *Z* > 3.09, *p* < .001). Four brightness levels also indicate the significance of individual bins, from the lighter (*Z* < 1.64, *p* > .05) to the darker (*Z* > 3.09, *p* < .001) level. While the expression-change response does not reach significance for any participant when emotions are expressed at low intensity, it progressively emerges at moderate intensity for some individuals in each group and reaches higher significance thresholds in healthy controls. The same is observed at high expression intensity despite a significant response for almost every participant

In order to determine whether the visual coding of facial expression is impaired in most 22q11DS individuals, significance of the expression-change response was also evaluated in every single participant (Fig. 3). No participants, both from the 22q11DS and control groups, exhibit a significant response for low-intensity facial expressions (all *Z*s < 1.41, all *p*s > .079) corroborating group-level observations. For emotions expressed at moderate intensity, 11 (i.e., 50%) participants with 22q11DS and 15 (i.e., 68%) healthy controls present with a significant expression-change response (*Z*s > 1.64, *p*s < .05). However, if we consider a higher significance threshold (i.e., *Z* > 2.32, *p* < .01), only 5 (i.e., 23%) individuals with 22q11DS still present with a significant response vs. 14 (i.e., 64%) healthy participants, thus demonstrating the reduced sensitivity of the visual system to brief expression changes in many 22q11DS individuals. An even higher threshold (i.e., *Z* > 3.09, *p* < .001) abolishes significant responses in the 22q11DS group compared with 10 (i.e., 45%) participants still remaining in the control group. For high-intensity facial expressions, 18 (i.e., 82%) participants with 22q11DS and all healthy controls show a significant expression-change response, but again *Z*-scores are lower in the 22q11DS group as clearly visible in Fig. 3.

### Reliability of the expression-change brain response

**Fig. 4:**
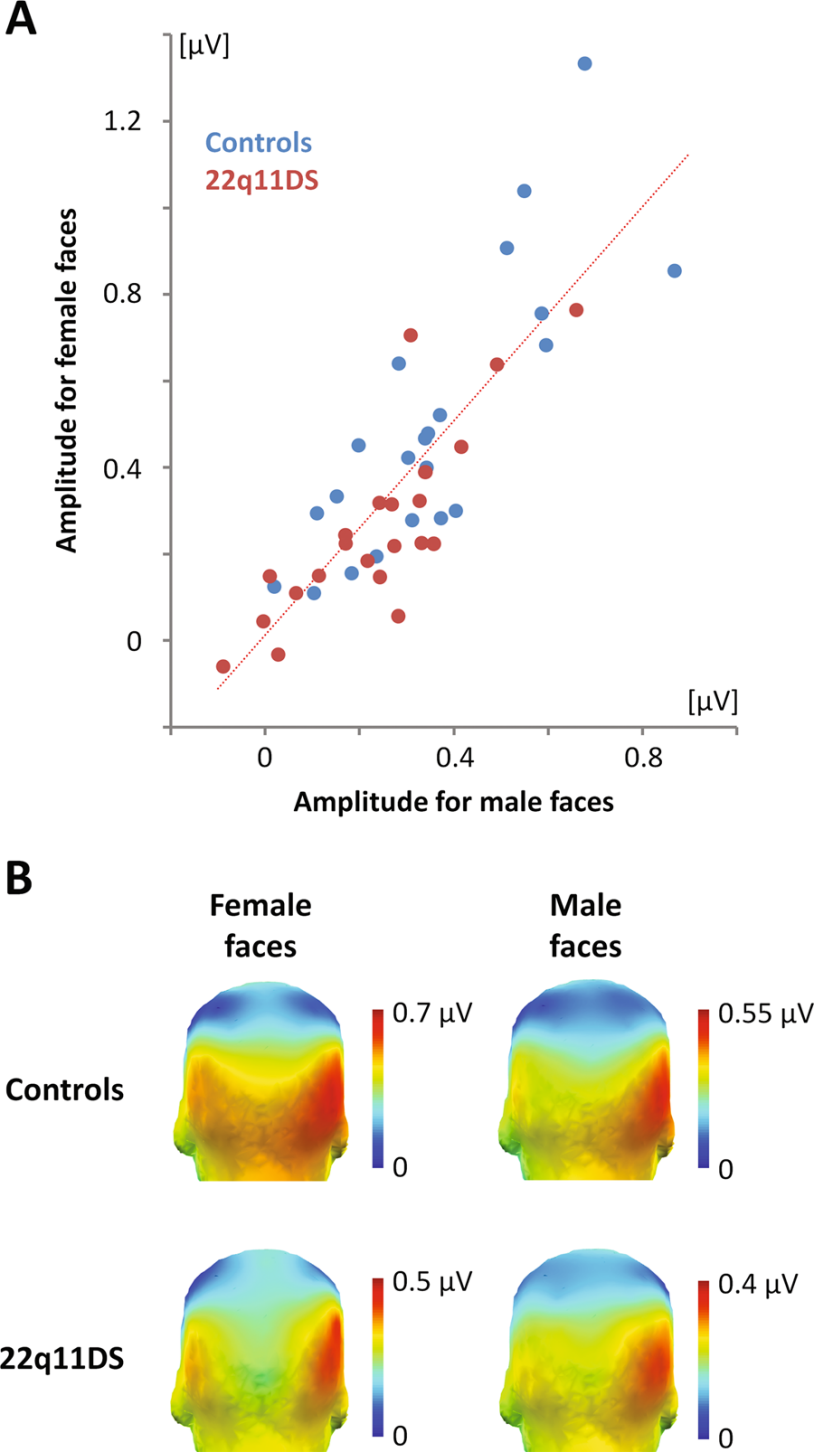
Reliability of the expression-change brain response. **a** Scatter plot showing that the strength of the expression-change response calculated between summed corrected amplitudes obtained for female and male faces and averaged across emotions, expression intensities, and channels (from the regions of interest) is highly reliable. 22q11DS participants are depicted in red while healthy controls are depicted in blue. **b** Three-dimensional topographical maps (posterior view) revealing that the topography of the expression-change response obtained for female (left) and male (right) faces and averaged across emotions and intensities for healthy controls (top) and 22q11DS participants (bottom) is also highly reliable. Note that color scales have been adapted for each map

The reliability of the expression-change response averaged across emotions and expression intensities was estimated by separating and correlating the amplitudes obtained for stimulation sequences using female and male faces. A first estimate was calculated with individual amplitudes averaged across channels included in the ROIs. It indicated that the strength of the response is highly reliable across measurements (Fig. 4a) for all participants (*r* = .83, *p* < .001) and separately for 22q11DS (*r* = .83, *p* < .001) and healthy (*r* = .81, *p* < .001) individuals. In addition, a second estimate was computed with the amplitude recorded for each channel over the scalp and averaged across participants. It revealed that the topography of the expression-change response is also highly reliable (Fig. 4b; all participants: *r* = .96, *p* < .001; 22q11DS: *r* = .90, *p* < .001; controls: *r* = .97, *p* < .001).

### Relationship to positive symptoms

**Fig. 5:**
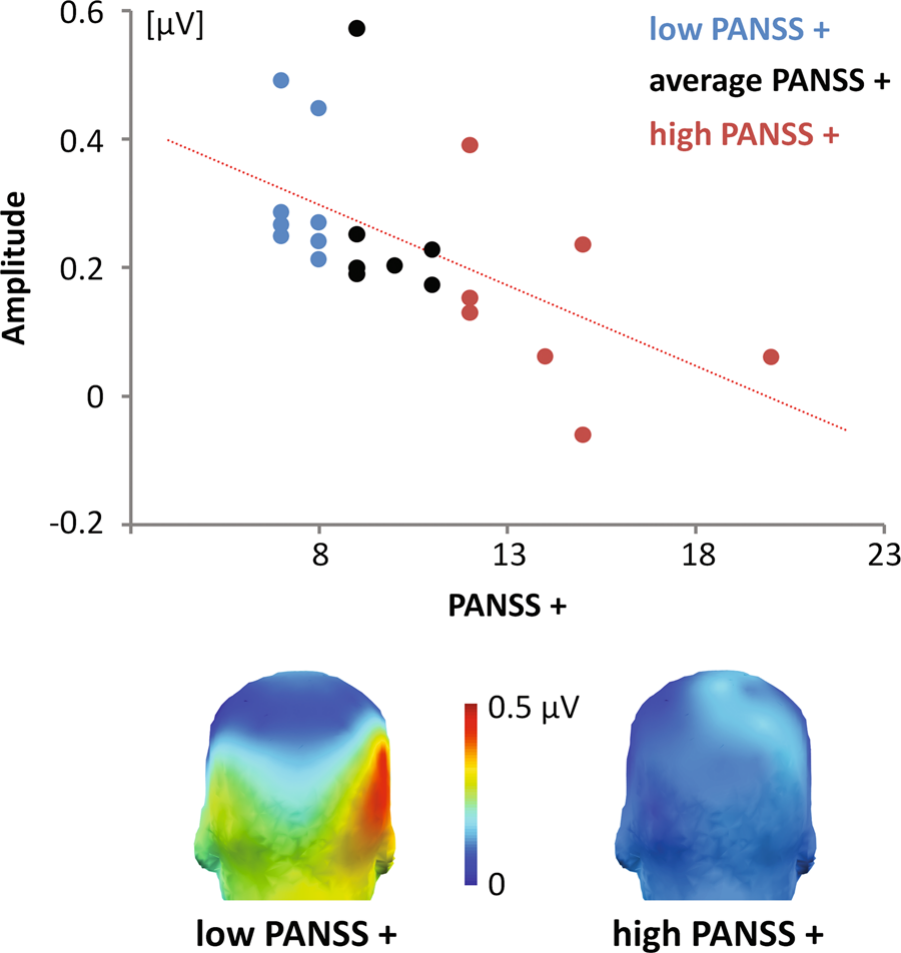
Association between the expression-change response and positive symptoms. Top: Scatter plot of the negative relationship between individual assessments of positive symptoms (PANSS+) in 22q11DS participants and summed corrected amplitudes of the expression-change response averaged across emotions, expression intensities and regions of interest (ROIs). Eight participants with the lowest PANSS+ scores are indicated in blue while seven participants with the highest scores are depicted in red. Individuals with intermediate scores are displayed in black. Bottom: Three-dimensional topographical maps (posterior view) of the expression-change response averaged across emotions and intensities for 22q11DS participants with low (left) and high (right) PANSS+ scores, showing the larger amplitude of the response in individuals with less positive symptoms

Correlations between the expression-change response averaged across emotions, expression intensities, and ROIs, and both clinical and neuropsychological ratings revealed a significant inverse association between the amplitude of the response and positive symptoms (*r* = −.59, *p* = .004). This indicates that the higher the neural response to expression changes is in 22q11DS participants, the lower are their positive symptoms (Fig. 5). No other significant correlations were found (all *p*s > .082) with clinical symptoms and cognitive processes. Correlations are fully detailed in Supplementary Results.

## Discussion

By using frequency-tagging with EEG, a highly effective approach for evaluation of visual perception^47^, the present study isolates a neural marker for impaired visual coding of facial expression in 22q11DS over occipito-temporal regions and quantifies this reduced brain response to brief changes of facial expression from a neutral face as about 64% of the “typical” response measured in healthy controls for emotions expressed at moderate and high intensities. This observation reinforces previous evidence about the difficulties of 22q11DS individuals for facial emotion processing^9,12–14,18–27^, and additionally clearly reveals that the impairment is subtended, at least partly, by a reduced visual sensitivity to changes of facial expression, as previously although indirectly suggested by neuroimaging findings^37–39^.

Most importantly for our purpose, the present observation provides several benefits in terms of social-cognitive assessment with critical implications for both researchers and clinicians. First, frequency-tagging in EEG is an objective approach thanks to pre-experimental definition of target frequencies. Here two brain responses were respectively tagged at 6 Hz and 1.2 Hz and directly quantified at these specific frequencies and their harmonics in the EEG frequency spectrum. This objectivity avoids variable analyses across studies (e.g., definition of time windows for event-related potential analysis in EEG studies), thus increasing reliability and favoring the development of standardized assessments in a clinical context.

Second, periodic stimulation associated with frequency-domain analysis provide high SNR measurement of the EEG signal captured in tiny target frequencies compared with non-periodic noise spreading to broad frequency ranges. Thanks to this high sensitivity, the brain response to expression changes was isolated in a few minutes of recording (i.e., only 100 s of stimulation for one emotion expressed at one intensity level), this short recording time allowing rapid evaluation. *Z*-scores were calculated using surrounding noise and provided direct significance estimation of the expression-change response in every individual participant. In addition, the expression-change response is highly reliable, both in terms of magnitude and topography. This key aspect of any approach aiming at individual testing would undeniably facilitate its translation into clinical practice. Admittedly, the approach must be consolidated for consistent individual measurements with high test–retest reliability determined at different testing times and high SNR responses in all individuals tested. Nonetheless, frequency-tagging in EEG is a promising tool to collect normative data directly from the brain in a large sample of healthy people, to define atypical visual coding of facial expression accordingly, and to apply individual assessment in any population.

Third, we provide a neural marker for automatic visual coding of facial expression that does not require any explicit response, weakening the contribution of response-related processes that can vary considerably across individuals and across development, and are easily contaminated by difficulties observed in 22q11DS^24,29–36^ and other disorders (e.g., verbal difficulties, reduced motivation and attention, impaired executive processes). As a consequence, contrary to previous studies that found a significant relationship between facial emotion recognition measured from behavior and other cognitive abilities^12,23,25^, the expression-change brain response measured here is unrelated to any cognitive ability evaluated during neuropsychological assessment. Moreover, frequency-tagging does not rely on post hoc subtraction^45,46^ to isolate the neural processes underlying facial expression perception from other unspecific processes. The expression-change response is a direct *differential* measure of the brain activity elicited by a change of facial expression from a neutral face exempt from other visual processes common to both neutral and expressive faces (e.g., low-level contrast-change detection) captured by the general visual response recorded at 6 Hz and harmonics. Accordingly, dissociated patterns were observed for the expression change vs. the general visual brain responses (see Supplementary Results for a detailed analysis of the latter and for the absence of significant correlation between the two responses). Altogether, these observations confirm that the expression-change response is a highly selective neural signature of automatic and implicit visual discrimination of facial expression giving the opportunity to test participants from various backgrounds without confounding perception with other cognitive skills. Hence, it might be used to differentiate various disorders that typically exhibit difficulties for facial expression recognition according to visual vs. non-visual impairments (e.g., post-perceptual difficulties in attributing emotions to clear-cut percepts; impaired explicit decision associated with reduced motivation, attention and executive functioning).

To a broader extent, frequency-tagging constitutes a unique approach for objective quantification of various cognitive functions in numerous populations whose condition precludes any classical neuropsychological assessment (e.g., high intellectual disability). Direct neural assessment might thus be used in a clinical context for diagnostic or even prognostic purpose. Similarly, the approach can easily explore a cognitive function across development. For instance, using the same design as in adults^53,54^, it was used for evaluating the development of reading abilities in preschool children^55^ and identified a neural marker of face categorization (i.e., vs. non-face objects) in 4- to 6-month-old infants^56^. For 22q11DS and other neurodevelopmental disorders already diagnosed in infancy, early biomarkers for the onset of later cognitive difficulties could be detected using the approach, offering high predictive value and opportunities to develop adequate remediation strategies for optimizing outcomes.

Ultimately, frequency-tagging can also contribute to the search for early biomarkers of psychotic symptoms and SSD and enhance diagnosis and early treatment strategies. Indeed, the brain response to expression change in 22q11DS is associated with positive symptoms of psychosis (and the general visual response with negative symptoms, Supplementary Results). While further studies should obviously consolidate these observations with a larger sample of participants and determine the developmental course of impaired facial expression perception in relation to psychosis onset, it holds high promise for future clinical interventions. It could be used in association with verbal IQ^31,32^ or executive processes^57,58^ also related to clinical symptoms of psychosis. However, verbal skills and executive functioning are late-maturing cognitive abilities in comparison with facial expression perception^59^. Hence, according to the opportunity of using frequency-tagging in childhood or infancy, the present signature of impaired visual processing of facial expression could serve as one of the most precocious biomarkers of later psychosis development.

In conclusion, frequency-tagging in EEG provides an implicit and reliable measure of the visual coding of facial expression that is obtained rapidly from brain activity and analyzed objectively (i.e., pre-experimentally defined analyses), with high SNR (i.e., signal captured in tiny target frequencies within high-resolution frequency spectrum), high selectivity (i.e., direct differential measure without post hoc subtraction), and large immunity from response-related processes (i.e., non-periodic orthogonal behavioral task). Thanks to this task-independent direct neural measurement of a cognitive process, the approach could be developed as a standardized tool for individual assessment of even difficult-to-test population at any age, opening avenues for both experimental exploration and clinical practice in the near future.

## Supplementary information

Supplementary Methods and Results

Supplementary Movie Description

Supplementary Movie
