# Supplementary material for: An implicit and reliable neural measure quantifying impaired visual coding of facial expression: evidence from the 22q11.2 deletion syndrome
Source: Transl Psychiatry. 2019 Feb 4;9:67. doi: 10.1038/s41398-019-0411-z (PMC6362075; doi:10.1038/s41398-019-0411-z)
Supplement: Supplementary file 1 — Supplementary Methods and Results [file 41398_2019_411_MOESM1_ESM.pdf]

An implicit and reliable neural measure quantifying impaired visual coding of facial expression: evidence from the 22q11.2 deletion syndrome

Supplementary Methods and Results

Supplementary Methods

Participants with 22q11DS

Supplementary Table S1. Sex, age and medication for each participant with 22q11DS at their inclusion in the study. In particular, 8 participants presented concomitant diagnosis of schizophrenia and were treated with antipsychotic medication. Anetholtri.: anetholtrithione, alimemaz.: alimemazine, alprazol.: alprazolam, aripipraz.: aripiprazole, citalop.: citalopram, escital.: escitalopram, fluoxet.: fluoxetine, haloper.: haloperidol, heptam.: heptaminol, hydroxyz.: hydroxyzine, loxap.: loxapine, levothyr.: levothyroxine, lormetaz.: lormetazepam, methylphe.: methylphenidate, mirtazap.: mirtazapine, olanzap.: olanzapine, paroxet.: paroxetine, risperid.: risperidone, sertal.: sertraline, tropatep.: tropatepine, zopiclo.: zopiclone.

22q11DS participant	Sex	Age	Medication				
			antipsychotic	antidepressant	anxiolytic	stimulant	others
Q01	F	28	N/A	N/A	N/A	N/A	N/A
Q02	M	25	haloper.	paroxet.	N/A	N/A	N/A
Q03	F	21	N/A	N/A	N/A	N/A	N/A
Q04	M	20	N/A	N/A	N/A	N/A	N/A
Q05	M	18	N/A	citalop.	N/A	methylphe.	N/A
Q06	M	17	risperid.	citalop.	hydroxyz.	methylphe.	heptam.<br>levothyr.
Q07	F	23	N/A	escital.	N/A	N/A	N/A
Q08	M	41	N/A	escital.	N/A	N/A	levothyr.
Q09	M	41	loxap.<br>olanzap.	N/A	N/A	N/A	zopiclo.
Q10	M	33	N/A	N/A	N/A	N/A	N/A
Q11	F	16	risperid.	sertral.	N/A	N/A	N/A
Q12	M	25	N/A	N/A	N/A	N/A	N/A
Q13	F	19	N/A	N/A	N/A	N/A	valproate
Q14	F	18	N/A	N/A	N/A	methylphe.	N/A
Q15	M	23	haloper.	mirtazap.	lormetaz.	N/A	tropatep.<br>anetholtri.<br>alimemaz.
Q16	M	40	aripipraz.	citalop.<br>mirtazap.	alprazol.	N/A	zopiclo.
Q17	F	20	N/A	fluoxet.	hydroxyz.	N/A	N/A
Q18	F	44	N/A	N/A	N/A	N/A	N/A
Q19	F	24	risperid.	escitalop.	alprazol.	N/A	N/A
Q20	M	20	N/A	N/A	N/A	N/A	N/A
Q21	F	20	N/A	N/A	N/A	N/A	N/A
Q22	M	20	aripipraz.	N/A	N/A	N/A	N/A

Neuropsychological assessment

Neuropsychological assessment evaluated basic visual functions (screening and incomplete letters tests from the visual object and space perception battery, VOSP[1]), visual object matching (minimal feature view test from the Birmingham object recognition battery, BORB[2]), visuospatial and perceptual abilities (Benton judgment of line orientation test, JLO[3]; copy of the Rey-Osterrieth complex figure, ROCF[4]), reading ability (fNART[5]), short- and long-term verbal memories (short- and long-term recalls from the California verbal learning test, CVLT[6]), working memory (forward and backward digit spans from the 4th edition of the Wechsler Adult Intelligence Scale, WAIS-IV[7]), processing speed (GZ index from the d2 test, d2-GZ[8]), and visual selective attention (KL index from the d2 test, d2-KL[8]). All tests are standardized and validated tools with high validity[1–3,5,6,9–11].

EEG data: preprocessing

All EEG analysis were carried out using Letswave 5 (http://www.nocions.org/letswave5) running on Matlab 2012 (MathWorks, USA) and largely followed analyses steps described in previous studies investigating facial expression perception using the same approach[12,13]. EEG data were first bandpass filtered (0.1–100 Hz) using a butterworth filter (4th order) and then down-sampled to 256 Hz to reduce file size and processing time. For each sequence, they were segmented in a 80-sec-long epoch according to 1 sec before the fade-in and 0.5 sec after the fade-out, thus resulting in 20 epochs corresponding to the 20 stimulation sequences tested throughout the experiment. An Independent Component Analysis (ICA) was computed[14] to remove components corresponding to eye blinks (recorded over prefrontal (Fp) channels) and muscular artifacts recorded over frontal and temporal electrodes. Channels with remaining noise or artifacts (i.e., containing modulations in amplitude exceeding ± 100 µV) were re-estimated using linear interpolation from the four neighboring electrodes (mean number across participants: 22q11DS: 3.1 channels, range: 0–11; controls: 2.2 channels, range: 0–11; no significant difference between the two groups, $T_{42} = 1.19$, $p = .24$). EEG data were then re-referenced to a common average reference.

EEG data: frequency-domain analysis

To increase signal-to-noise ratio, the four preprocessed epochs obtained for each emotion (i.e., one per individual face) were averaged resulting in five 80-sec epochs (i.e., one per emotion). They were each segmented in three shorter epochs corresponding to the three 25-sec periods (i.e., one per intensity step) within each stimulation sequence. Each epoch began with the presentation of the first change of expression (i.e., 0.667 sec, 25.667 secs and 50.667 secs after the fade-in respectively for the low, moderate and high expression intensities) and lasted 25 secs (i.e., exactly thirty 1.2 Hz cycles, 6400 time bins). Frequency-domain amplitude spectra were then extracted with a frequency resolution of 1/25 = 0.04 Hz by applying a fast Fourier transform (FFT). Thanks to this high resolution, 29 frequency bins were extracted between two target frequencies (e.g., between 1.2 and 2.4 Hz, see below) allowing unambiguous identification of the brain responses elicited by the 6 Hz rapid stimulation and the 1.2 Hz expression-changes, and noise estimation at each frequency bin.

Since noise amplitude varies across the EEG frequency spectrum, being higher at lower frequencies and locally higher in certain frequency bands (e.g., in the 8–12 Hz alpha band), it was estimated at each frequency bin using the 20 surrounding frequency bins (10 on each side), excluding the immediately adjacent and the two most extreme (i.e., minimum and maximum) bins (noise range ≈ 0.96 Hz). Using this noise estimation, we first computed signal-to-noise ratios (SNR) by dividing the amplitude at each frequency bin by the mean amplitude of the noise. Contrary to FFT amplitude spectra, SNR reflect the strength of the signal at all frequency ranges irrespective of its absolute amplitude. Hence, SNR were used at the group level (for both 22q11DS and control participants) to visually inspect the responses recorded at both the 6 Hz base rate of stimulation and the 1.2 Hz of expression-change and their harmonics (i.e., integer multiples) for each expression intensity averaged across emotions (Supplementary Figure S2 in Supplementary Results).

To quantify each brain response (i.e., the general visual response elicited by the 6 Hz base rate of rapid stimulation, and the expression-change response elicited by the 1.2 Hz expression changes) in a single value, several harmonics can be combined[15]. Hence, we determined how many harmonics were significant for both general (6 Hz) and expression-change (1.2 Hz) responses. Since the expression-change response is expected to be nearly absent for the low-intensity expressions, with a progressive increase in amplitude with increasing intensity[13], harmonics significance was estimated by only considering EEG data obtained for emotions expressed at high intensity. FFT amplitude spectra were grand-averaged across participants (irrespective of their group), electrodes and emotions, and *Z*-scores were calculated at each frequency bin by subtracting the mean noise amplitude (using the same noise definition as for SNR computations) and dividing by the standard deviation of the noise. Harmonics were considered significant until the *Z*-scores for two consecutive harmonics were no longer greater than 1.64 ($p < .05$, one-tailed, signal > noise). Significant harmonics (Supplementary Table S2) were found until the 7th harmonic (i.e., 42 Hz) for the general visual response, and until the 14th harmonic (i.e., 16.8 Hz) for the expression-change response.

Supplementary Table S2. *Z*-scores calculated for each harmonic of both the general and the expression-change visual responses on grand-averaged FFT data across participants, electrodes and emotions expressed at high intensity. Harmonics were further considered until two consecutive harmonics were no longer significant (i.e., $Z > 1.64$, $p < .05$, one-tailed, signal > noise). Significant *Z*-scores are indicated in bold and the corresponding frequency is indicated for each harmonic of each response. Harmonics corresponding to the general visual response (e.g., 5th harmonic = 6 Hz) were not considered for the expression-change response.

Harmonic	General visual response	Expression-change response
1st	6 Hz: **139.48**	1.2 Hz: **1.91**
2nd	12 Hz: **48.94**	2.4 Hz: **5.70**
3rd	18 Hz: **33.01**	3.6 Hz: **11.94**
4th	24 Hz: **10.03**	4.8 Hz: **21.68**
5th	30 Hz: **7.90**	
6th	36 Hz: **3.53**	7.2 Hz: **4.65**
7th	42 Hz: **2.57**	8.4 Hz: **5.19**
8th	48 Hz: 0.57	9.6 Hz: **2.05**
9th	54 Hz: -0.11	10.8 Hz: 0.68
10th		
11th		13.2 Hz: **2.20**
12th		14.4 Hz: **5.99**
13th		15.6 Hz: 1.14
14th		16.8 Hz: **3.78**
15th		
16th		19.2 Hz: 0.66
17th		20.4 Hz: -1.08

Both general and expression-change visual responses were summarized in a single value by summing FFT data for significant harmonics (excluding the 5th (i.e., 6 Hz) and the 10th (i.e., 12 Hz) harmonics for the expression-change response which correspond to the general response). These summed amplitudes were used to calculate *Z*-scores estimating the overall significance of each response across significant channels depending on the group of participants and the experimental condition (e.g., for each expression intensity, see ‘Results’ for such analysis with the expression-change response and Supplementary Results below for the general visual response).

Z-scores calculated on summed amplitudes were also used as a data-driven approach to determine, for each brain response, different regions-of-interest (ROIs) combining several electrodes for further statistical analyses. For the general visual response, ROIs were defined using the grand-averaged data across participants and all experimental conditions since its topography is not expected to differ between emotions or expression intensities[12,13]. In contrast, different topographies between emotions were previously described for the expression-change response[12,13]. ROIs were thus determined separately for each emotion using the grand-averaged data across participants and expression intensities so that any differences between emotions in further analyses would not be explained by topographical differences.

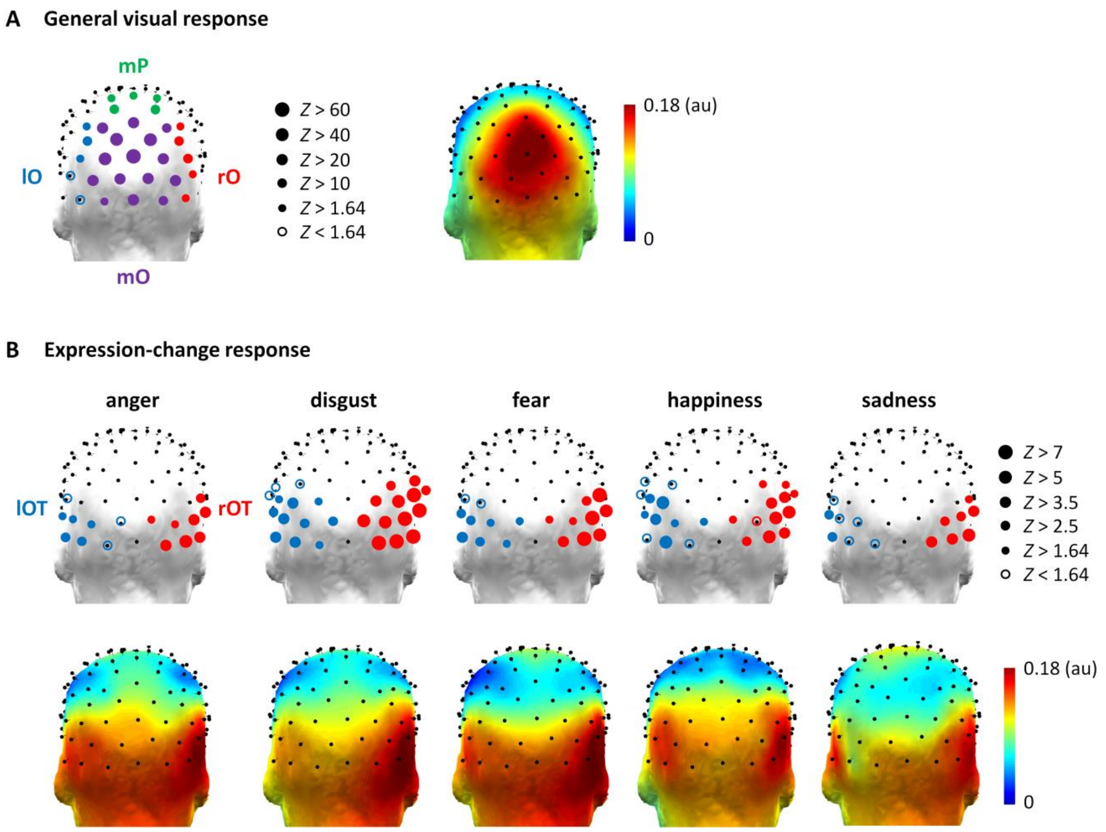

Supplementary Figure S1. 3D-topographical maps (posterior view) of both the electrodes reaching significance ($Z > 1.64$, one-tailed, signal > noise) for the definition of the regions-of-interest (ROIs) and of normalized summed corrected amplitudes (color maps in arbitrary unit [au]) showing the spatial distribution of each brain response over the scalp. For the maps illustrating ROIs, bubble size at every single channel reflects the strength of its *Z*-score. Unfilled bubbles indicate non-significant lateral electrodes included in ROIs to match their symmetrical significant electrodes in the other hemisphere (mainly left channels matching right channels). **A.** For the general visual response, EEG data are averaged across all participants and all experimental conditions. Significant channels (left) largely encompass the medial occipital and parietal regions and were pooled in four ROIs based on the visual inspection of the response (right). ROIs were defined according to medial (purple), left (blue) and right (red) occipital sites (mO, lO, rO), and a medial parietal site (mP, green). **B.** For the expression-change response, EEG data are averaged across all participants and expression intensities for each emotion. Significant channels (top) are more laterally distributed than for the general visual response, with an advantage for the right hemisphere clearly visible in the representations of the response (bottom). Two ROIs were defined according to left and right occipito-temporal regions (lOT, rOT). As revealed by the different channels pooled in both ROIs, topographies differ according to the emotion expressed. In particular, disgust presents with a more largely distributed response compared with the other emotions, and sadness with a more restricted response.

Z-scores were computed a first time to determine which electrodes over the scalp showed a significant response. For the general visual response, all electrodes largely reach significance with the lowest *Z*-score found for the parietal channel CCP6h (Z = 45.54) and the highest for the occipital channel Oz (Z = 253.27). For the expression-change response, the number of electrodes reaching significance depends on the emotion expressed, from 63 significant channels for sadness to 115 for disgust. The occipito-temporal channel PPO10h presents the highest *Z*-score for all emotions (from Z = 3.95 for anger to Z = 7.61 for disgust) except for sadness (occipito-temporal channel P10: Z = 4.00).

Since many electrodes are significant for both responses, we scaled their amplitude differences on scalp-wide magnitude to isolate the main electrodes contributing to each response. We normalized summed amplitudes at each significant channel by dividing by the square root of the sum of squared amplitudes of all significant channels[16]. Then, *Z*-scores were calculated a second time on these normalized amplitudes to identify the remaining electrodes showing a significant response. Finally, based on visual inspection of both responses, these electrodes were pooled in different ROIs for each response. For the general visual response, four ROIs were defined according to medial, left and right occipital sites (mO, lO, rO), and a medial parietal region (mP). For the expression-change response, two ROIs were determined for each emotion according to left and right occipito-temporal sites (lOT, rOT). In other words, the two same ROIs were further analyzed for the expression-change response but the electrodes included in these ROIs differed according to the emotion expressed. For lateral ROIs (i.e., lO and rO, lOT and rOT), symmetrical electrodes across hemispheres were always included (i.e., if an electrode reached significance in one hemisphere but not its symmetrical electrode in the other hemisphere, the latter was nonetheless included). The electrodes reaching significance for each response and their respective ROIs are displayed in Supplementary Figure S1. In addition, Supplementary Figure S1 displays 3D-topographical maps of normalized summed corrected amplitudes (see below) to illustrate the correspondence between ROIs and the spatial distribution of each response over the scalp.

Both responses were quantified in a single value expressed in microvolt (µV). FFT amplitude spectra were first corrected by subtracting the mean noise amplitude (using the same noise definition as for SNR and *Z*-scores) at each frequency bin and corrected amplitudes were summed across significant harmonics for each brain response. Summed corrected amplitudes were computed for each participant and condition for statistical analyses and grand-averaged across participants and/or conditions for illustration purpose. Hence, thanks to noise subtraction, corrected amplitudes directly quantify the magnitude of each response (i.e., they are not different from zero if the response is absent). Statistical analyses were conducted for each brain response using *Z*-scores (see above) and repeated-measures ANOVAs with *Group* (22q11DS, controls) as a between-subject factor, and *Emotion* (anger, disgust, fear, happiness, sadness), *Intensity* (low, moderate, high) and *ROI* (4 *vs*. 2 levels respectively for the general and expression-change visual responses, see above) as within-subject factors (see 'Methods and materials' for further details).

Finally, the reliability of the expression-change response was estimated. For each participant, the 20 preprocessed 80-sec-long EEG segments (see 'EEG data: preprocessing') were split between those using female and male faces (i.e., 10 segments each) and averaged in the time-domain across emotions resulting in two 80-sec epochs (i.e., 1 per gender). They were segmented in shorter epochs lasting 75 secs (i.e., exactly ninety 1.2 Hz cycles) from the presentation of the first change of expression (i.e., 0.667 sec after the fade-in). An FFT was applied (frequency resolution: 1/75 = 0.013 Hz) and the expression-change response was quantified from its summed corrected amplitude across harmonics (see above). A first estimate of reliability was conducted by averaging the response across all channels included in the ROIs for at least one emotion, resulting in one value per participant. A second estimate was conducted by averaging the response across participants, resulting in one value per channel (for the 64 channels over the scalp). For both estimates, Pearson's correlation coefficient was calculated between the responses obtained for female and male sequences. The first coefficient thus estimates whether the strength of the response is reliable, while the second coefficient estimates whether the topography of the response is reliable.

Supplementary Results

Clinical and neuropsychological assessments

Results for clinical and neuropsychological assessments (Supplementary Table S3) revealed that around 15% of participants with 22q11DS have several cognitive alterations encompassing verbal memory (CVLT), working memory (digit spans), processing speed (d2-GZ) and selective attention (d2-KL). Most interestingly, they show that while people with 22q11DS do not exhibit any deficit in low-level visual functions (VOSP) and visual object matching (BORB-MFW), they are impaired for higher-level visual functions such as visuospatial and perceptual abilities (JLO, around 40% of participants; ROCF, around 80%). However, please note that this last conclusion is only tentative because ROCF is also often used to evaluate executive functioning in addition to visual skills. The present observations may thus partly reflect difficulties in executive processes.

Supplementary Table S3. Mean score, standard deviation (SD) and range for raw scores obtained by 22q11DS participants for each clinical and neuropsychological assessment. The number of participants with atypical scores (*N* atypical) based on normative data cited in Methods and Materials is also indicated for neuropsychological tests. For VOSP, BORB and JLO, atypical scores are determined by a cutoff value (below 15 for VOSP screening, below 17 for VOSP incomplete letters, below 16 for BORB-MFW, and below 19 for JLO). For ROCF, CVLT, digit spans and d2, *Z*-scores were calculated and atypical scores correspond to *Z*-scores < -1.96 ($p < .05$, two-tailed). For fNART, scores are already converted in IQ scores (atypical below 70). PANSS: positive and negative symptoms scale, VOSP: visual object and space perception battery, BORB-MFW: minimal feature view test from the Birmingham object recognition battery, JLO: Benton Judgment of Line Orientation test, ROCF: copy of the Rey-Osterrieth complex figure, fNART: French National Adult Reading Test, CVLT: California verbal learning test, WAIS-IV: 4th edition of the Wechsler Adult Intelligence Scale.

Test	Mean ± SD	Range	*N* atypical
PANSS			
positive symptoms	10.36 ± 3.33	7–20	N/A
negative symptoms	15.24 ± 6.01	9–31	N/A
general psychopathology	31.45 ± 8.77	18–52	N/A
VOSP			
screening	19.55 ± 0.67	18–20	0
incomplete letters	19.41 ± 0.79	18–20	0
BORB-MFW	24.41 ± 1.14	20–25	0
JLO	18.86 ± 5.85	4–27	9
ROCF	25.64 ± 7.50	2–35	18
fNART	98.02 ± 6.34	87–115	0
CVLT			
short-term recall	8.86 ± 3.38	0–12	4
long-term recall	9.67 ± 3.71	0–15	3
WAIS-IV digit span			
forward	5.23 ± 1.15	3–7	4
backward	4.05 ± 1.17	2–6	1
d2			
GZ (processing speed)	382.76 ± 106.65	138–563	5
KL (visual selective attention)	137.33 ± 47.16	31–231	2

Orthogonal behavioral task

Both participants with 22q11DS (*M* = 446 ± 11 (*SEM*) ms) and healthy controls (*M* = 448 ± 13 ms) rapidly detected the circle-to-square shape-change of the fixation throughout the experiment, with mean accuracy near ceiling for 22q11DS (*M* = 93.0 ± 1.4%) and healthy individuals (*M* = 95.2 ± 1.9%). Repeated-measures ANOVAs run on individual response times and accuracies with *Emotion* (anger, disgust, fear, happiness, sadness) and *Intensity* (low, moderate, high) as within-subject factors, and *Group* (22q11DS, controls) as a between-subject factor confirmed equivalent performance between groups of participants irrespective of the experimental condition since neither the main effect of *Group* (response times: $F_{1,\ 42}$ = .02, p = .90; accuracy: $F_{1,\ 42}$ = .86, p = .36) nor any other effects involving the *Group* factor (response times: all *p*s > .14; accuracy: all *p*s > .20) reached significance for both measures. In sum, both groups of participants efficiently performed the orthogonal behavioral task throughout the experiment. This indicates that they both paid full attention to the screen during the fast periodic visual stimulation, and that this simple task easily achieved by individuals with 22q11DS cannot account for differences between groups in EEG data.

EEG data: signal-to-noise ratios (SNR) for both brain responses and their harmonics

For both groups of participants, the 6 Hz base rate of rapid visual stimulation elicited synchronized periodic EEG activities of high SNR at the same frequency and its harmonics (i.e., integer multiples; e.g., 12 Hz, 18 Hz) mainly over medial occipital regions (i.e., signal around 2 to 8 times larger than the noise over the electrode Oz for harmonics displayed in Supplementary Figure S2). These activities reflect the general sensitivity of the visual system to all cues rapidly changing at 6 Hz (e.g., contrast).

Most interestingly, as clearly visible in Supplementary Figure S2, introducing brief 1.2 Hz periodic changes of emotional expression from neutrality at low expression intensity does not elicit identifiable EEG responses (i.e., SNR ≈ 1, signal ≈ noise) while synchronized brain activities at 1.2 Hz and its harmonics progressively appear as expression intensity increases. These responses selectively reflecting the visual processing of the periodic expression-changes are mainly recorded over right occipito-temporal regions for both groups of participants with the signal around 1.5 to 2.5 times larger than the noise over the electrode PPO10h (Supplementary Figure S2) for harmonics ranging from 1.2 to 7.2 Hz (higher harmonics are not clearly identified by visual inspection but nonetheless significant, see Supplementary Table S2). Importantly for our purpose, expression-change selective activities seem lower in 22q11DS participants than healthy controls, suggesting impaired visual coding of facial expression in individuals with 22q11DS.

EEG data: summed corrected amplitudes for the general visual response

As in previous studies using similar stimulation parameters[12,13], the general visual response is centered over medial occipital regions (Supplementary Figure S3) with larger amplitudes over mO (*M* = 1.27 ± .10 µV) than the other ROIs (lO: *M* = .82 ± .08 µV, rO: *M* = .91 ± .08 µV, mP: *M* = .75 ± .08 µV, *ROI* effect: $F_{2.5,\ 103.6}$ = 19.55, ε = .82, p < .001, η_p^2 = .32). The general visual response reflects the brain synchronization to the rapid stream of stimulation. It captures a mixture of low- and higher-level processes elicited by all cues rapidly changing at 6 Hz and common to both neutral and expressive faces (e.g., low-level processes detecting luminance- and contrast-changes). Hence, contrary to the expression-change response (see 'EEG data: summed corrected amplitudes for the expression-change response depending on the emotion expressed by the face'), the general visual response is not modulated by the emotion expressed by the face (*Emotion* effect: $F_{4,\ 168}$ = 1.19, p = .32) and is clearly identified throughout the stimulation sequence (all *Z*s > 123.79, all *p*s < .001 for all expression intensities and for both groups of participants). As in Leleu and collaborators' study[13], it is even larger for emotions expressed at low (*M* = 1.02 ± .08 µV) than moderate (*M* = .90 ± .07 µV) and high (*M* = .89 ± .07 µV) intensities (*Intensity* effect: $F_{1.4,\ 60.5}$ = 38.86, ε = .72, p < .001, η_p^2 = .48) for an overall decrease of 13.3%. Since expression intensity varied along the stimulation sequence, it reveals that the general visual response was larger at the start of the sequence and further

decreased, in contrast with the expression-change response which was absent at the start of the sequence and then progressively appeared as a function of expression intensity (see 'Results'). This opposite pattern shows that the two brain responses reflect distinct neural processes. In particular, the slight decrease of the general visual response during the first 25 secs. of stimulation is probably explained by adaptation to visual cues repeating at 6 Hz (e.g., facial identity[17,18] and/or by reduced attention to the stimulation[19,20]), but it is certainly unrelated to the intensity of the brief changes of facial expression.

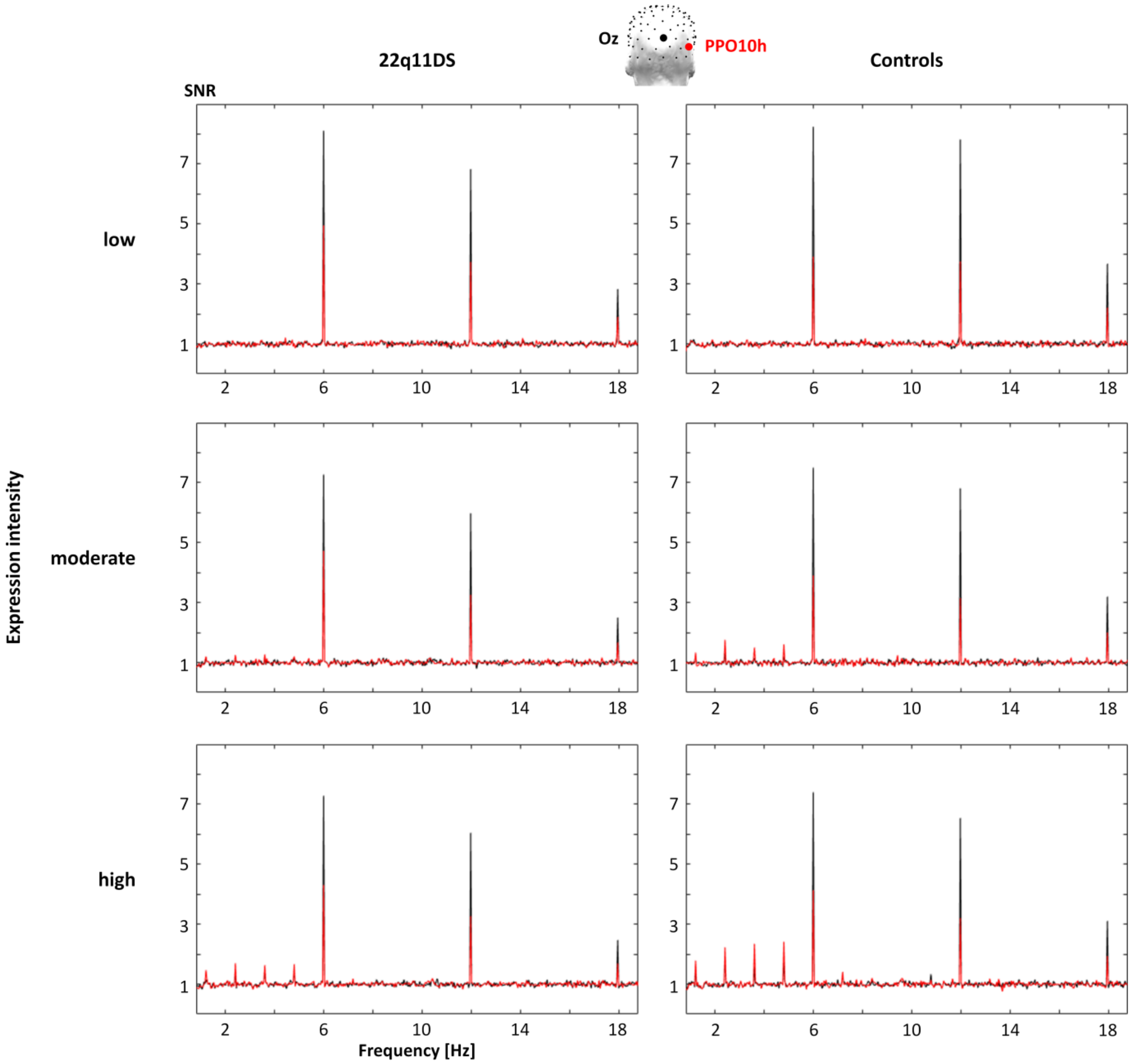

Supplementary Figure S2. Signal-to-noise ratios (SNR) calculated on the FFT amplitude spectra averaged across emotions for both 22q11DS individuals (left) and healthy controls (right) and for low (top), moderate (middle) and high (bottom) expression intensities. SNR are displayed over the medial occipital channel Oz (black) and the right occipito-temporal channel PPO10h (red). Target frequencies (i.e., harmonics of the 1.2 Hz and 6 Hz frequencies) can be identified until 18 Hz but harmonics are significant until 42 Hz for the response elicited at the 6 Hz rate of rapid stimulation (Supplementary Table S2). For this response, high SNR activities are clearly visible for all expression intensities and for both groups of participants mainly over channel Oz, reflecting the general sensitivity of the visual system to all cues rapidly changing at 6 Hz. In contrast, EEG activities at 1.2 Hz and harmonics reflecting the visual coding of the brief expression-changes are not identifiable for emotions expressed at low intensity (SNR ≈ 1, signal ≈ noise) and progressively appear as intensity increases, mainly over channel PPO10h. Importantly, while these activities are visible for both groups of participants, their SNR is lower in 22q11DS individuals than in healthy controls.

Interestingly, differences between groups of participants revealed another dissociation between the two brain responses with a significant main effect of *Group* ($F_{1, 42}$ = 4.98, p = .031, η_p^2 = .11) for the general visual response (Supplementary Figure S3) due to larger amplitudes for individuals with 22q11DS (M = 1.09 ± .11 µV) than healthy controls (M = .78 ± .07 µV). Hence, while participants with 22q11DS present a poorer neural sensitivity to brief expression-changes than healthy individuals, their visual system is rather more sensitive to all cues rapidly alternating at 6 Hz with a benefit of about 38% of the response observed for control participants. However, this effect did not survive when controlling for the effects of medications (see 'Effects of medications and age on both brain responses'). No other effects involving the *Group* factor were found for the general visual response (all *p*s > .10).

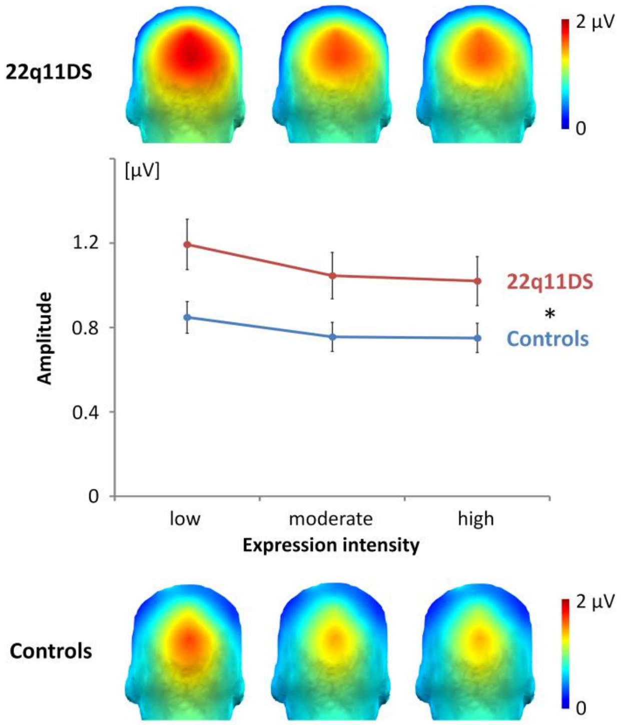

Supplementary Figure S3. 3-D topographical maps (posterior view) of summed corrected amplitudes of the general visual response as a function of expression intensity averaged across emotions for both 22q11DS individuals (top) and healthy controls (bottom). Center: the same data displayed averaged across regions-of-interest (ROIs). Bars represent standard errors of the mean. Data are displayed for each group and each expression intensity, but only the main effects of *Group* (* p < .05) and *Intensity* were significant and did not interact. The general visual response is clearly identified for both groups of participants and decreases during the 25 first secs. of the stimulation sequence (i.e., when emotions are expressed at low intensity). However, it was significantly larger in 22q11DS participants.

EEG data: summed corrected amplitudes for the expression-change response depending on the emotion expressed by the face

As in previous studies[12,13], the magnitude of the expression-change response varies according to the emotion expressed (*Emotion* effect: $F_{4, 168}$ = 11.83, p < .001, η_p^2 = .22) with larger amplitudes for disgust (M = .41 ± .05 µV), and lower amplitudes for sadness (M = .18 ± .03 µV) than for the other emotions (anger: M = .28 ± .04 µV, fear: M = .34 ± .03 µV, happiness: M = .30 ± .03 µV). Interestingly, a significant *Group* × *Emotion* interaction ($F_{4, 168}$ = 2.65, p = .035, η_p^2 = .06) revealed variable patterns of amplitude differences between groups depending on the facial emotion expressed (Supplementary Figure S4). The response is significantly lower in 22q11DS individuals than healthy

controls for anger (22q11DS: $M = .20 \pm .04$ µV, controls: $M = .36 \pm .05$ µV, $p = .023$), disgust (22q11DS: $M = .29 \pm .06$ µV, controls: $M = .53 \pm .07$ µV, $p = .014$) and sadness (22q11DS: $M = .12 \pm .03$ µV, controls: $M = .23 \pm .04$ µV, $p = .035$), but not for fear (22q11DS: $M = .31 \pm .05$ µV, controls: $M = .37 \pm .05$ µV, $p = .45$) and happiness (22q11DS: $M = .27 \pm .05$ µV, controls: $M = .33 \pm .05$ µV, $p = .41$). Hence, the expression-change response to angry, disgusted and sad faces is reduced by approximately 45% compared with healthy individuals while a non-significant reduction of about 15% is observed for fear and happiness.

This observation is in line with previous studies showing spared processing for fear[21,22] or happiness[23–26]. Here, the emotions expressed by the face do not have to be explicitly recognized and named. Hence, the observed reduced neural sensitivity is unrelated to a post-perceptual difficulty in attributing emotions to clear-cut percepts, but rather points to less sharply-defined visual representations of facial expressions in 22q11DS[26]. The larger impairment for angry, disgusted and sad faces may thus be explained by their close configurations of facial movements, leading to higher confusions[27], compared with more distinguishable expressions of fear and happiness. However, this variable visual sensitivity to changes of facial expression across emotion categories in 22q11DS must be considered with caution and further investigated since the *Group* × *Emotion* interaction did not survive when controlling for the effects of antidepressant and antipsychotic medications (see 'Effects of medications and age on both brain responses').

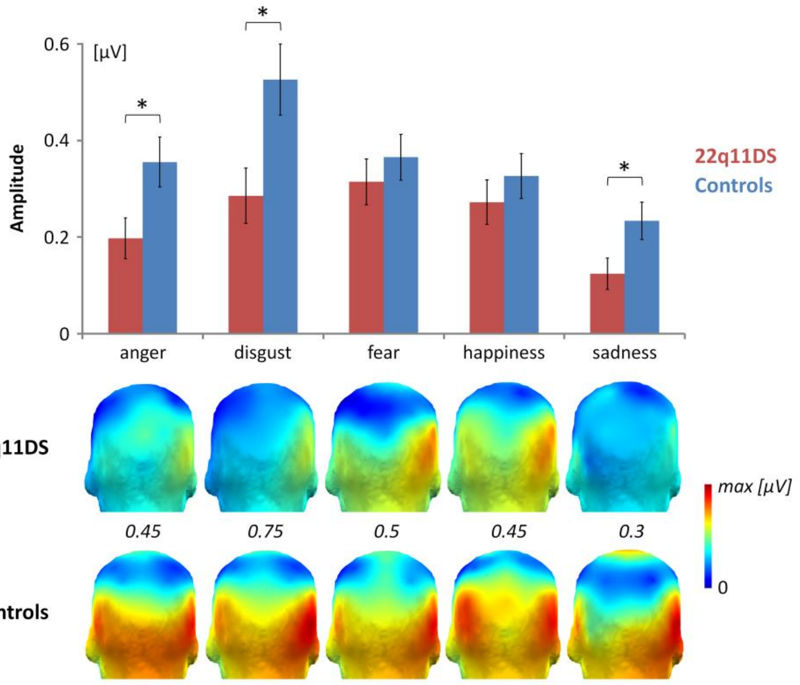

Supplementary Figure S4. Top: summed corrected amplitudes of the expression-change response averaged across expression intensities and regions-of-interest (ROIs) for each emotion and for both 22q11DS individuals and healthy controls. Bars represent standard errors of the mean. (* $p < .05$). Bottom: 3-D topographical maps (posterior view) of the same data. Note that color-coded scales vary according to the emotion expressed with the maximal value for each emotion indicated in italic between the maps from each group of participant. The expression-change response is significantly lower in 22q11DS for anger, disgust and sadness with a reduction of about 45% of the response observed for healthy controls. However, this effect did not survive when controlling for the effect of both antidepressant and antipsychotic medications (see 'Effects of medications and age on both brain responses').

Correlations between each brain response and clinical and neuropsychological assessments

Pearson's correlations were calculated between both brain responses quantified from summed corrected amplitudes averaged across emotions, expression intensities and ROIs, and both clinical and neuropsychological ratings (Supplementary Table S4). As detailed in the Results section, the expression-change response is inversely correlated with positive symptoms. A marginally significant association between its amplitude and visuospatial and perceptual abilities as evaluated by ROCF was also observed. For the general visual response, a significant inverse relationship was found with negative symptoms. In addition, marginally significant correlations were observed with positive symptoms, visuospatial and perceptual abilities estimated by ROCF, and working memory evaluated from backward digit span of the WAIS IV. No other significant associations were identified.

Supplementary Table S4. Pearson's correlations between each clinical and neuropsychological assessment (see Supplementary Table S3 for the definition of acronyms) and each brain response averaged across emotions, expression intensities and regions-of-interest (ROIs). Significant correlations are depicted in bold (* $p < .05$; ** $p < .01$) and *p* values are indicated next to coefficients for marginally significant ($p < .10$) correlations. The expression-change response was negatively associated with positive symptoms while the general visual response was negatively associated with negative symptoms. Other marginally significant relationships were observed, in particular between visuospatial and perceptual abilities evaluated by ROCF and both brain responses.

Test		Expression-change response	General visual response
PANSS			
	positive symptoms	**-.59****	$-.037^{.09}$
	negative symptoms	-.08	**-.51***
	general psychopathology	-.07	-.19
VOSP			
	screening	-.22	-.32
	incomplete letters	-.07	.32
BORB-MFW		-.05	.36
JLO		.14	.10
ROCF		$.38^{.08}$	$.40^{06}$
fNART		.30	.09
CVLT			
	short-term recall	.02	.04
	long-term recall	.08	.12
WAIS-IV digit span			
	forward	-.02	-.12
	backward	.14	$.36^{09}$
d2			
	GZ (processing speed)	.01	-.19
	KL (visual selective attention)	.18	-.26

Correlation between the expression-change and the general visual responses

Finally, we tested the association between the two brain responses and found no significant correlation ($r = .23$, $p = .30$). Hence, there is no direct relationship between the low amplitude expression-change response *vs*. the high amplitude general visual response observed in individuals with 22q11DS. This confirms the dissociation between the two visual responses which capture distinct neural processes within the same stimulation sequence.

Effects of medications and age on both brain responses

Since more than a third of participants with 22q11DS were treated with antipsychotic (N = 8, 36%) and/or antidepressant (N = 10, 45%) medications (Supplementary Table S1), we evaluated whether these treatments may explain differences across 22q11DS participants for both brain responses. Comparisons between 22q11DS individuals treated with antipsychotic medication and those untreated did not reveal any significant difference for both the expression-change response (treated: M = .22 ± .03 µV, untreated: M = .25 ± .04 µV, T_{20} = .57, p = .58) and the general visual response (treated: M = 1.17 ± .24 µV, untreated: M = 1.04 ± .12 µV, T_{20} = .53, p = .60). In contrast, while no significant difference (T_{20} = 1.47, p = .16) emerged between participants treated with antidepressant medication (M = .29 ± .05 µV) and those untreated (M = .20 ± .04 µV) for the expression-change response, a marginally significant effect (T_{20} = 2.02, p = .057) was found for the general visual response with a larger amplitude for treated (M = 1.32 ± .20 µV) compared with untreated (M = .89 ± .11 µV) 22q11DS individuals (Supplementary Figure S5). The high-amplitude general visual response observed for the 22q11DS group compared with the control group (see 'EEG data: summed corrected amplitudes for the general visual response' and Supplementary Figure S3) may thus be induced, at least partly, by antidepressant medication.

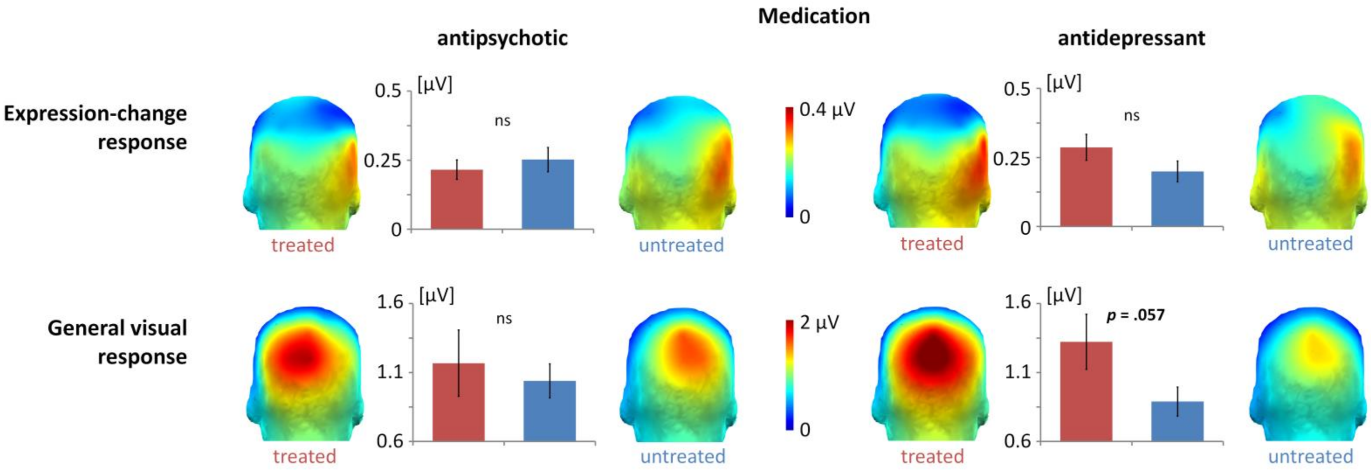

Supplementary Figure S5. 3-D topographical maps (posterior view) of summed corrected amplitudes of both the expression-change (top) and the general visual (bottom) responses averaged across emotions and expression intensities for treated and untreated 22q11DS individuals with antipsychotic (left) and antidepressant (right) medications. Between 'treated' and 'untreated' maps, the same data are displayed averaged across regions-of-interest (ROIs). Bars represent standard errors of the mean. Treatments do not have any effect on each brain response (ns: p > .10) except a marginally significant (p = .057) influence of antidepressant medication on the general visual response with a larger response observed for treated *vs*. untreated 22q11DS participants (bottom right). This suggests that the greater amplitude of the general visual response found for 22q11DS compared with healthy participants (see 'EEG data: summed corrected amplitudes for the general visual response' and Supplementary Figure S3) may be induced, at least partly, by antidepressant medication.

To further confirm the effects of medications on both brain responses, and to determine whether the large age range across participants may drive response differences, we finally ran an ANCOVA for each brain response using the same factors as in previous ANOVAs and adding *Antipsychotic* and *Antidepressant Treatments* (treated, untreated) as categorical predictors, and *Age* as a continuous predictor. For the expression-change response, no main effects of predictors reached significance (all ps > .12) and the main effect of *Group* ($F_{1, 39}$ = 4.46, p = .041, η_p^2 = .10) as well as the *Group* × *Intensity* interaction ($F_{1.3, 52.5}$ = 4.89, ε = .67, p = .022, η_p^2 = .11) were still significant (see

‘Results’). In contrast, the *Group* × *Emotion* interaction disappeared ($F_{4, 156}$ = 1.73, p = .15). It slightly increased after removing the contribution of *Antidepressant Treatment* ($F_{4, 156}$ = 1.93, p = .11) and reached significance when additionally removing the contribution of *Antipsychotic Treatment* ($F_{4, 156}$ = 2.61, p = .037, η_p^2 = .06). For the general visual response, an effect of *Antidepressant Treatment* ($F_{1, 39}$ = 5.14, p = .029, η_p^2 = .12) was observed, and the main effect of *Group* ($F_{1, 39}$ = .56, p = .46) did not reach significance anymore. When removing the contribution of *Antidepressant Treatment*, the effect of Group strongly increased ($F_{1, 40}$ = 2.59, p = .12) but was still non-significant. It only reached significance ($F_{1, 41}$ = 4.76, p = .035, η_p^2 = .10) after removing the contribution of *Antipsychotic Treatment*.

In sum, results for both brain responses were unaffected by the age of participants. In addition, while the reduced expression-change response for 22q11DS individuals compared with heathy controls is not explained by any treatment, they both explain the variable pattern of group differences depending on the emotion expressed by the brief changes of facial expression. Likewise, medications largely drive the high-amplitude general visual response observed for 22q11DS participants, in particular antidepressant treatment, and thus explain the difference between groups of participants that disappears when controlling for their effects.

Supplementary References

1 Warrington EK, James M. *The Visual Object and Space Perception Battery*. Thames Valley Test Company: Bury St. Edmunds, UK, 1991.
2 Riddoch MJ, Humphreys GW. *Birmingham object recognition battery*. Lawrence Erlbaum Associates, 1993.
3 Benton AL, Hamsher KD, Varney NR, Spreen O. *Judgment of line orientation*. Oxford University Press: New York, 1983.
4 Wallon P, Mesmin C. *Test de la figure complexe de Rey*. ECPA: Paris, 2009.
5 Mackinnon A, Mulligan R. Estimation de l'intelligence prémorbide chez les francophones. *L'Encéphale* 2005; **31**: 31–43.
6 Delis DC, Freeland J, Kramer JH, Kaplan E. Integrating clinical assessment with cognitive neuroscience: construct validation of the California Verbal Learning Test. *J Consult Clin Psychol* 1988; **56**: 123–130.
7 Wechsler D. *WAIS IV - Nouvelle version de l'échelle d'intelligence de Wechsler pour adultes - Quatrième édition*. ECPA: Paris, 2011.
8 Brickenkamp R. *Test d'attention concentrée-d2*. ECPA: Paris, 1998.
9 Hamby SL, Wilkins JW, Barry NS. Organizational quality on the Rey-Osterrieth and Taylor Complex Figure Tests: A new scoring system. *Psychol Assess* 1993; **5**: 27.
10 Wechsler D. Wechsler adult intelligence scale–Fourth Edition (WAIS–IV). *San Antonio Tex Psychol Corp* 2008.
11 Bates ME, Lemay EP. The d2 Test of Attention: Construct validity and extensions in scoring techniques. *J Int Neuropsychol Soc* 2004; **10**: 392–400.
12 Dzhelyova M, Jacques C, Rossion B. At a Single Glance: Fast Periodic Visual Stimulation Uncovers the Spatio-Temporal Dynamics of Brief Facial Expression Changes in the Human Brain. *Cereb Cortex* 2017; **27**: 4106–4123.
13 Leleu A, Dzhelyova M, Rossion B, Brochard R, Durand K, Schaal B *et al.* Tuning functions for automatic detection of brief changes of facial expression in the human brain. *NeuroImage* 2018; **179**: 235–251.
14 Makeig S, Bell AJ, Jung T-P, Sejnowski TJ. Independent component analysis of electroencephalographic data. In: D. Touretzky, M. Mozer, M. Hasselmo (eds). *Advances in neural information processing systems*. MIT Press: Cambridge, MA, 1996, pp 145–151.
15 Retter TL, Rossion B. Uncovering the neural magnitude and spatio-temporal dynamics of natural image categorization in a fast visual stream. *Neuropsychologia* 2016; **91**: 9–28.
16 McCarthy G, Wood CC. Scalp distributions of event-related potentials: An ambiguity associated with analysis of variance models. *Electroencephalogr Clin Neurophysiol Potentials Sect* 1985; **62**: 203–208.
17 Nemrodov D, Jacques C, Rossion B. Temporal dynamics of repetition suppression to individual faces presented at a fast periodic rate. *Int J Psychophysiol* 2015; **98**: 35–43.
18 Retter TL, Rossion B. Visual adaptation provides objective electrophysiological evidence of facial identity discrimination. *Cortex* 2016; **80**: 35–50.
19 Kim YJ, Grabowecky M, Paller KA, Muthu K, Suzuki S. Attention induces synchronization-based response gain in steady-state visual evoked potentials. *Nat Neurosci* 2007; **10**: 117–125.
20 Morgan ST, Hansen JC, Hillyard SA. Selective attention to stimulus location modulates the steady-state visual evoked potential. *Proc Natl Acad Sci* 1996; **93**: 4770–4774.
21 Jalbrzikowski M, Carter C, Senturk D, Chow C, Hopkins JM, Green MF *et al.* Social cognition in 22q11.2 microdeletion syndrome: Relevance to psychosis? *Schizophr Res* 2012; **142**: 99–107.
22 Franchini M, Schaer M, Glaser B, Kott-Radecka M, Debanné M, Schneider M *et al.* Visual processing of emotional dynamic faces in 22q11.2 deletion syndrome. *J Intellect Disabil Res* 2016; **60**: 308–321.
23 Campbell LE, McCabe K, Leadbeater K, Schall U, Loughland C, Rich D. Visual scanning of faces in 22q11.2 deletion syndrome: Attention to the mouth or the eyes? *Psychiatry Res* 2010; **177**: 211–215.
24 McCabe KL, Rich D, Loughland CM, Schall U, Campbell LE. Visual scanpath abnormalities in 22q11.2 deletion syndrome: Is this a face specific deficit? *Psychiatry Res* 2011; **189**: 292–298.
25 McCabe KL, Melville JL, Rich D, Strutt PA, Cooper G, Loughland CM *et al.* Divergent patterns of social cognition performance in autism and 22q11. 2 deletion syndrome (22q11DS). *J Autism Dev Disord* 2013; **43**: 1926–1934.
26 Leleu A, Saucourt G, Rigard C, Chesnoy G, Baudouin J-Y, Rossi M *et al.* Facial emotion perception by intensity in children and adolescents with 22q11.2 deletion syndrome. *Eur Child Adolesc Psychiatry* 2016; **25**: 297–310.
27 Tottenham N, Tanaka JW, Leon AC, McCarry T, Nurse M, Hare TA *et al.* The NimStim set of facial expressions: Judgments from untrained research participants. *Psychiatry Res* 2009; **168**: 242–249.
