# Supplementary material for: An implicit and reliable neural measure quantifying impaired visual coding of facial expression: evidence from the 22q11.2 deletion syndrome
Source: Transl Psychiatry. 2019 Feb 4;9:67. doi: 10.1038/s41398-019-0411-z (PMC6362075; doi:10.1038/s41398-019-0411-z)
Supplement: Supplementary file 2 — Supplementary Movie Description [file 41398_2019_411_MOESM2_ESM.pdf]

Supplementary Movie illustrates the fast periodic visual stimulation used in the study.
